# Protein kinase C-delta inactivation inhibits the proliferation and survival of cancer stem cells in culture and *in vivo*

**DOI:** 10.1186/1471-2407-14-90

**Published:** 2014-02-14

**Authors:** Zhihong Chen, Lora W Forman, Robert M Williams, Douglas V Faller

**Affiliations:** 1 Cancer Center, Boston University School of Medicine, K-712C, 72 E. Concord St., Boston, MA 02118, USA; 2 Department of Medicine, Boston University School of Medicine, K-712C, 72 E. Concord St., Boston, MA 02118, USA; 3 Department of Chemistry, Colorado State University, 1301 Centre Ave, Fort Collins, CO 80523, USA; 4 University of Colorado Cancer Center, Aurora, CO 80045, USA; 5 Department of Pediatrics, Boston University School of Medicine, K-712C, 72 E. Concord St., Boston, MA 02118, USA; 6 Department of Biochemistry, Boston University School of Medicine, K-712C, 72 E. Concord St., Boston, MA 02118, USA; 7 Department of Microbiology, Boston University School of Medicine, K-712C, 72 E. Concord St., Boston, MA 02118, USA; 8 Department of Pathology, Boston University School of Medicine, K-712C, 72 E. Concord St., Boston, MA 02118, USA; 9 Department of Laboratory Medicine, Boston University School of Medicine, K-712C, 72 E. Concord St., Boston, MA 02118, USA

**Keywords:** Protein Kinase C isozymes, Synthetic lethal interaction, Cancer-initiating cell, Xenograft tumor model

## Abstract

**Background:**

A subpopulation of tumor cells with distinct stem-like properties (cancer stem-like cells, CSCs) may be responsible for tumor initiation, invasive growth, and possibly dissemination to distant organ sites. CSCs exhibit a spectrum of biological, biochemical, and molecular features that are consistent with a stem-like phenotype, including growth as non-adherent spheres (clonogenic potential), ability to form a new tumor in xenograft assays, unlimited self-renewal, and the capacity for multipotency and lineage-specific differentiation. PKCδ is a novel class serine/threonine kinase of the PKC family, and functions in a number of cellular activities including cell proliferation, survival or apoptosis. PKCδ has previously been validated as a synthetic lethal target in cancer cells of multiple types with aberrant activation of Ras signaling, using both genetic (shRNA and dominant-negative PKCδ mutants) and small molecule inhibitors. In contrast, PKCδ is not required for the proliferation or survival of normal cells, suggesting the potential tumor-specificity of a PKCδ-targeted approach.

**Methods:**

shRNA knockdown was used validate PKCδ as a target in primary cancer stem cell lines and stem-like cells derived from human tumor cell lines, including breast, pancreatic, prostate and melanoma tumor cells. Novel and potent small molecule PKCδ inhibitors were employed in assays monitoring apoptosis, proliferation and clonogenic capacity of these cancer stem-like populations. Significant differences among data sets were determined using two-tailed Student’s t tests or ANOVA.

**Results:**

We demonstrate that CSC-like populations derived from multiple types of human primary tumors, from human cancer cell lines, and from transformed human cells, require PKCδ activity and are susceptible to agents which deplete PKCδ protein or activity. Inhibition of PKCδ by specific genetic strategies (shRNA) or by novel small molecule inhibitors is growth inhibitory and cytotoxic to multiple types of human CSCs in culture. PKCδ inhibition efficiently prevents tumor sphere outgrowth from tumor cell cultures, with exposure times as short as six hours. Small-molecule PKCδ inhibitors also inhibit human CSC growth *in vivo* in a mouse xenograft model.

**Conclusions:**

These findings suggest that the novel PKC isozyme PKCδ may represent a new molecular target for cancer stem cell populations.

## Background

Much recent data supports the model that a subpopulation of tumor cells with distinct stem-like properties is responsible for tumor initiation, invasive growth, and possibly dissemination to distant organ sites [1-3]. This small subpopulation of cells can divide asymmetrically, producing an identical daughter cell and a more differentiated cell, which, during their subsequent divisions, generate the vast majority of tumor bulk [4,5]. A number of names have been used to identify this subpopulation, including “cancer progenitor cells,” “cancer stem cell-like cells,” and “cancer-initiating cells,” but the term “cancer stem cell” (CSC) has received wide acceptance [6].

The first identification of CSCs in solid tumors was made in 2003, when CSCs were identified and isolated from breast cancers using CD44 and CD24 markers [7]. Subsequently, CSCs have been identified in a variety of solid tumors, including glioblastoma [8-10], osteosarcoma [11], chondrosarcoma [12], prostate cancer [13], ovarian cancer [14-18], gastric cancer [19], lung cancer [20,21], colon cancer [22-25], pancreatic cancer [26,27], melanoma [28-30], head and neck cancer [31], and others. CSCs isolated from these different tumor types share some common characteristics including drug resistance, ability to repopulate tumors, and asymmetric division.

CSC exhibit a spectrum of biological, biochemical, and molecular features that are consistent with a stem-like phenotype, including growth as non-adherent spheres (clonogenic potential), superior ability to form a new tumor in *in vivo* xenograft assays, unlimited self-renewal, and the capacity for multipotency and lineage-specific differentiation [1,32-35]. In particular, CSCs are able to form colonies from a single cell more efficiently than their progeny [36] and to grow as spheres in non-adherent, serum-free culture conditions [37]. Sphere formation in non-adherent cultures has been used as a surrogate *in vitro* method for detecting CSCs from primary human tumors [8,20,25,38,39]. CSC populations also variably exhibit “stem cell-like” markers, such as Nanog, Sox2, aldehyde-dehydrogenase positivity, and telomerase.

Chemoresistance is also considered a hallmark of CSCs [6,40]. They characteristically survive chemo- and radio-therapeutic interventions [41] and may thus be responsible for both tumor relapse and metastasis [42]. CSCs are often innately less sensitive to treatment than are the bulk of the tumor cells that they generate [43,44]. These features support the hypothesis that CSCs are the cell subpopulation that is most likely responsible for treatment failure and cancer recurrence [32].

Aberrant activation of Ras signaling, either through mutation of the Ras genes themselves, or through constitutive upstream or downstream signaling, is very common in solid tumors. We have previously identified the protein kinase C delta (PKCδ) isozyme as a Ras synthetic lethal interactor [45-48]. PKCδ is a serine/threonine kinase of the PKC family, a member of the novel class, and functions in a number of cellular activities including cell proliferation, survival or apoptosis [49]. However, PKCδ is not required for the proliferation of normal cells, and PKCδ-null animals develop normally and are fertile, suggesting the potential tumor-specificity of a PKCδ-targeted approach [50]. PKCδ was validated as a target in cancer cells of multiple types with aberrant activation of Ras signaling, using both genetic (siRNA and dominant-negative PKCδ) and small molecule inhibitors [45], by our group [45,47] and later by others [51,52]. “Ras-dependency” in these tumors was not required for these synthetic-lethal cytotoxic effects [45,46]. Tumors with aberrant activation of the PI_3_K pathway or the Raf-MEK-ERK pathway in the setting of wild-type RAS alleles have also been shown to require PKCδ activity for proliferation or survival [47,48].

In this report, we demonstrate that CSC-like cell populations derived from multiple types of human primary tumors, from human cancer cell lines, and from transformed human cells require PKCδ activity and are susceptible to agents which deplete PKCδ protein or activity.

## Methods

### Cell culture

MCF10A and MCF10C breast cell lines were derived at the Barbara Ann Karmanos Cancer Institute (Detroit, MI) and maintained in DMEM-F/12 medium containing 5% heat-inactivated horse serum, 10 μg/mL insulin, 20 ng/mL epidermal growth factor, 0.1 μg/mL cholera enterotoxin, and 0.5 μg/mL hydrocortisone [53,54]. Breast cancer cell lines MCF7, Hs587T, and MDA231 were purchased from ATCC, and were propagated in 10% fetal bovine serum (Invitrogen, Grand Island, NY); Dulbecco’s Modification of Earle’s Media (Cellgro, Herndon, VA); 2 mM L-Glutamine (Invitrogen); 200 U Penicillin/ml; 200 μg Streptomycin/ml (Invitrogen).

Human breast cancer stem cells (BCSC: CD133+, CD44+, SSEA3/4+, Oct4+, Alkaline Phosphatase+, Aldehyde Dehydrogenase+, Telomerase+), pancreatic cancer stem cells (PCSC: CD44^+^, CD133^+^, SSEA3/4^+^, Oct4^+^, Alkaline Phosphatase^+^, Aldehyde Dehydrogenase^+^, Telomerase^+^, and Nestin^+^), and prostate cancer stem cells (PrCSC: CD44^+^, CD133^+^, SSEA3/4^+^, Oct4^+^, alkaline phosphatase^+^, aldehyde dehydrogenase^+^, and telomerase^+^) were purchased from Celprogen (San Pedro, CA), and cultured using specialized media and tissue culture plastic and matrix, to preserve their CSC phenotype, according to the manufacturer’s instructions.

### Reagents

Rottlerin was purchased from (EMD Biosciences, San Diego, CA). The PKCδ inhibitor KAM1 was previously described [47]. BJE6-106 was synthesized as described elsewhere [55]. Briefly, 9-(2-(trifluoro-λ^4^-boranyl)ethyl)-9*H*-carbazole, potassium salt (Molander Salt 1), 6-bromo-2,2-dimethyl-2H-chromene-8-carbaldehyde, 64.0 mg (0.213 mmol, 1 equiv.), PdCl2(dppf)-CH2Cl2, and anhydrous Cs_2_CO_3_ were combined to form 6-(2-(9*H*-carbazol-9-yl)ethyl)-2,2-dimethyl-2*H*-chromene-8-carbaldehyde (BJE6-106).

### Tumor sphere formation

Tumor self-renewing and anchorage-independent spheroids were obtained by culturing breast cancer cells MCF7, Hs587T and MDA231; melanoma cells SBcl2 and FM6; human breast cancer stem cells and pancreatic cancer stem cells in stem cell-selective conditions according to the manufacturer’s instructions (StemCell Technologies, Tukwila, WA). Briefly, cancer and cancer stem cells were propagated in 6-well ultra-low adherent plates (Corning) in Complete MammoCult Medium (Human) by adding 50 mL of MammoCult Proliferation Supplements to 450 mL of MammoCult Basal Medium (StemCell Technologies). The following were added to obtain Complete MammoCult Medium: 4 ug/mL Heparin (StemCell Technologies), 0.48 μg/mL hydrocortisone (StemCell Technologies), 200 U penicillin/ml; and 200 μg streptomycin/ml (Invitrogen).

### Flow cytometry

Cell staining for CD24 or CD44: MCF7 and MCF7 spheres, Hs587T and Hs587T spheres, MDA231 and MDA231 spheres, breast cancer stem cells and breast cancer stem cell spheres were collected and stained or dual-stained with Fluorescein isothiocyanate (FITC)-anti-CD24 and (PerCP-Cy)-anti-CD44 (BD Pharmingen, San Diego, CA) monoclonal antibody (mAbs) for 30 min on ice. The stained cancer cells and sphere populations were analyzed by FACSCAN analysis.

### Clonogenic assays

100,000 cells were seeded on 100 mm dishes with 10 ml media per dish [47]. On day 4, cells were treated with a PKCδ inhibitor or vehicle control for either 6, 18, 24 or 48 hours. Cells were trypsinized; counted *via* the trypan blue exclusion method in order to determine the number of live cells in the sample, and 300 live cells were seeded in triplicate onto 6-well plates. Cells were monitored for appropriate colony size and re-fed every three to four days. At Day 15, cells were stained with ethidium bromide [56] and counted using UVP LabWorks software (Waltham, MA).

### Cell proliferation assays

Cell proliferation was assessed using an MTT [3-(4,5-dimethylthiazol-2-yl)-2,5-diphenyltetrazolium bromide] assay (Roche, Mannheim, Germany). The number of viable cells growing in a single well on a 96-well microtiter plate was estimated by adding 10 μl of MTT solution (5 mg/ml in phosphate-buffered saline [PBS]). After 4 h of incubation at 37°C, the stain was diluted with 100 μl of dimethyl sulfoxide. The optical densities were quantified at a test wavelength of 570 nm and a reference wavelength of 690 nm on a multiwell spectrophotometer. In some assays, MTS was used as substrate (Promega, Madison, WI), and the absorbance of the product was monitored at 490 nm. Cell enumeration was carried out using a hemocytometer, and viable cells identified by trypan blue exclusion.

### PKC kinase activity assays

Assays were carried out using recombinant PKCδ or PKCα, (Invitrogen) and the Z-lyte Kinase Assays (Invitrogen) with a “PKC-kinase-specific” peptide substrate. FRET interactions produce a change in fluorescence (ex455/ex520) upon phosphorylation. The kit was used according to the manufacturer’s instructions.

### Cytotoxicity assay

LDH release was assessed by spectrophotometrically measuring the oxidation of NADH in both the cells and media. Cells were seeded in 24-well plates, and exposed to PKCδ inhibitors or vehicle. After different times of exposure, cytotoxicity was quantified by a standard measurement of LDH release with the use of the LDH assay kit (Roche Molecular Biochemicals) according to the manufacturer’s protocol. Briefly, total culture medium was cleared by centrifugation. For assay of released LDH, supernatants were collected. To assess total LDH in cells, Triton X-100 was added to vehicle (control) wells to release intracellular LDH. LDH assay reagent was added to lysates or supernatants and incubated for up to 30 min at room temperature in dark, the reaction was stopped, and the absorbance was measured at 490 nm. The percentage of LDH release was then calculated as the LDH in the supernatants as a fraction of the total LDH.

### Immunoblot analyses

Levels of proteins were measured and quantitated in cells as we have previously reported [45]. Harvested cells were disrupted in a buffer containing 20 mM Tris (pH 7.4), 0.5% NP-40, and 250 mM NaCl with protease and phosphatase inhibitors. Total protein (40 μg) was separated on 10% SDS-polyacrylamide gels and transferred to nitrocellulose membranes or PVDF membranes. Membranes were blocked overnight and probed with affinity-purified antibodies against: PKCδ (BD Transduction Labs, San Jose, CA), or β-actin or α-tubulin (Sigma Aldrich, St. Louis, MO). Antibodies against human ERK, phospho-ERK1/2 (Thr202/Tyr204), AKT and phospho-AKT (Ser473), JNK and phospho-JNK (Thr183/Tyr185) were purchased from Cell Signaling (Danvers, MA). After washing, the blots were incubated with horseradish peroxidase-conjugated secondary antibodies and visualized using the Amersham enhanced chemiluminescence ECL system, and quantitated by digital densitometry.

### Down-regulation of PKC by shRNA and lentiviral vectors

shRNA duplexes for PKCδ (shRNAs) were obtained from Qiagen (Valencia, Ca). The shRNA sequences for targeting PKCδ and the corresponding scrambled shRNAs used as negative controls were previously described [47]. The lentiviral vectors were previously described [46]. After infection of cells with the vectors, one aliquot was utilized in proliferation assays and a parallel aliquot was subjected to immunoblotting to assay the efficiency of the knockdown.

### Xenograft studies

These studies were performed with the approval of the Institutional Animal Care and Use Committee of Boston University. Breast cancer stem cells (2 × 10^5^) grown from a metastatic tumor were suspended in human breast cancer stem cell complete growth media (Celprogen, San Pedro, CA) and injected subcutaneous into the right flank of female J:NU mice (The Jackson Laboratory, ME) under anesthesia. After palpable tumors developed, the mice were divided into two groups of animals. The control group received daily intraperitoneal injections of vehicle (DMSO) while the treatment group received daily intraperitoneal injections of a PKCδ inhibitor (rottlerin 5,000 μg/kg) for 15 days. The length and width of tumors were measured with a vernier caliper and tumor volumes were calculated. Survival was calculated as the day tumors reached the maximum size allowed by the protocol (2 cm diameter).

### Statistical analysis

Experiments were carried out in triplicate for all experimental conditions. Data are shown as mean ± SD. Where applicable, a two-tailed Student’s t test or ANOVA was performed on the means of two sets of sample data and considered significant if p ≤ 0.05.

## Results

### Inhibition of PKCδ is growth-inhibitory and cytotoxic in human prostate and pancreatic cancer stem cells

**Figure 1:**
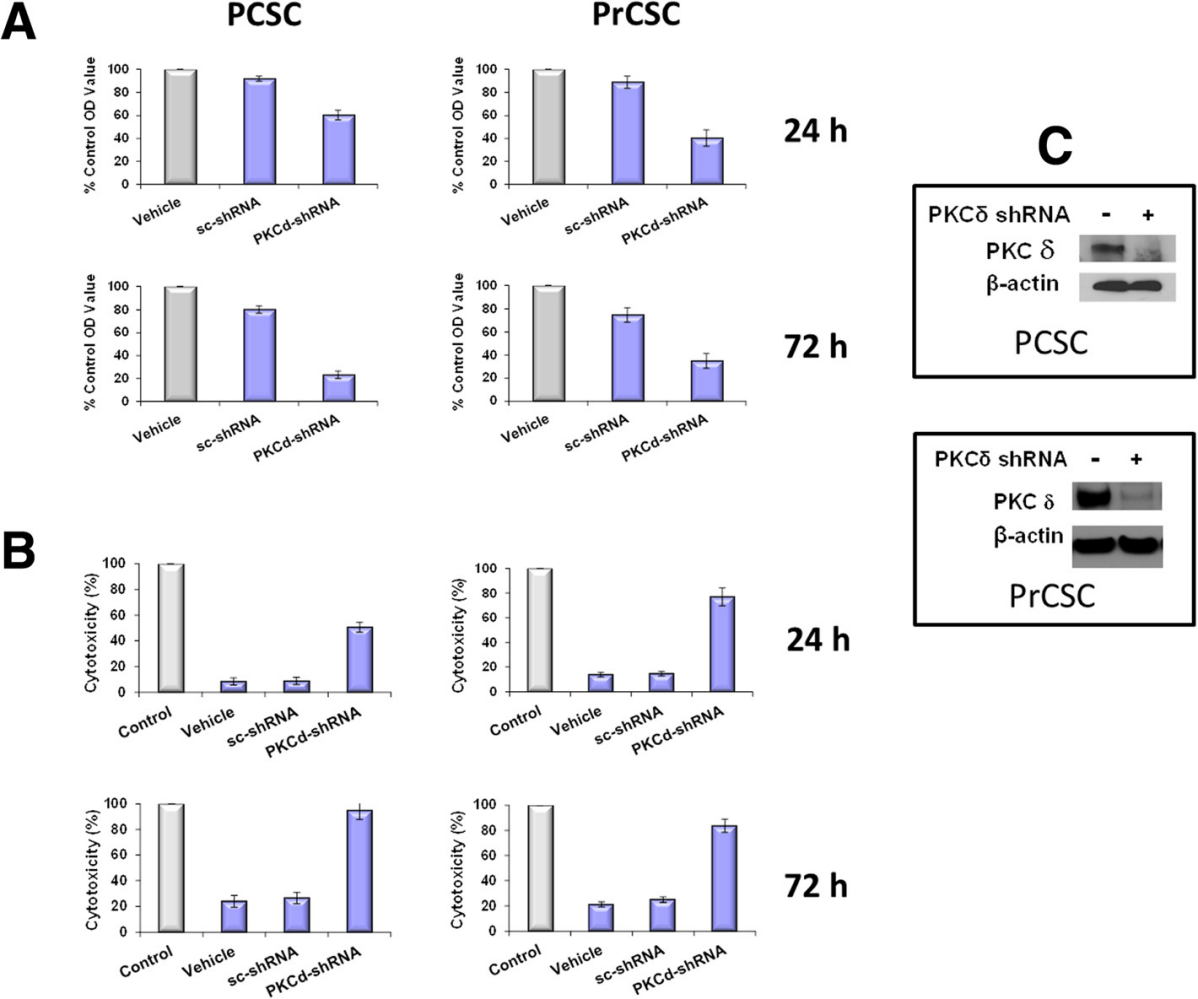
**Effects of PKCδ knockdown by shRNA on proliferation and viability of human pancreatic (PCSC) and prostate (PrCSC) cancer stem cell cultures. (A)** PCSC and PrCSC cells were grown to 50% confluence in 96-well plates and then infected with PKCδ-shRNA-expressing lentivirus vector or a lentiviral vector containing a scrambled shRNA (sc-shRNA). The corresponding equivalent volumes of diluent used for infection served as vehicle controls (Vehicle). 24 and 72 hr after transfection, cell mass was evaluated by MTS assay. Error bars represent SEM. p values for comparison between control (scrambled shRNA) and PKCδ-shRNA effects on cell number reached significance at 24 hr of exposure (p < 0.001) for all cell lines, and remained significant at the 72 hr time point. **(B)** PCSC and PrCSC cells were grown to 50% confluence in 96-well plates and then infected with PKCδ-shRNA or scrambled shRNA (sc-shRNA) expressing lentiviruses. The corresponding equivalent volumes of diluent were used as vehicle controls (Vehicle). After 24 and 72 hr of infection, cell cytotoxicity was evaluated by LDH-release assay. Total maximal LDH release was assigned the arbitrary value of 100% (Control). Error bars represent SEM. p values for comparison between effects on LDH release for cells infected with scrambled shRNA-expressing vectors compared to PKCδ-shRNA vectors reached significance at 24 hr of exposure (p < 0.01) for all cell lines, and remained significant at the 72 hr time point. **(C)** Immunoblot analysis of PKCδ protein levels in the same cell lines 72 hr after infection with PKCδ-targeting shRNA expressing lentiviral vectors (+) or scrambled shRNA (-). PKCδ-targeted shRNA vectors efficiently reduced PKCδ protein expression. Immunoblotting with a β-actin antibody after stripping the blots served as a loading control.

The sensitivity of human cancer stem cell cultures to inhibition of PKCδ was first examined using shRNA methodology to specifically and selectively knockdown transcripts for this PKC isozyme and thereby specifically validate PKCδ as a target in CSCs. Cell cultures derived from a primary human pancreatic adenocarcinoma (PCSC) and from a primary human prostate adenocarcinoma (PrCSC), isolated by phenotypic markers, were studied. These cells were characterized as “stem-like” by a number of criteria. The PCSC and the PrCSC cultures were CD44^+^, CD133^+^, Nanog^+^, Sox2^+^, aldehyde dehydrogenase^+^, and telomerase^+^. The PCSC cultures were also Nestin^+^. Both cell types were tumorigenic at <1000 cells in xenograft assays in SCID mice, and also formed tumor spheroids at high efficiency. Lentiviral vectors expressing PKCδ-specific shRNAs (PKCδ-shRNA), which we have previously shown to be specific for the PKCδ isozyme among all the other PKC isozymes [45-47], were used to deplete PKCδ levels in the cells. A vector containing a scrambled shRNA (sc-shRNA) served as a control. Specific knockdown of PKCδ by shRNA was growth-inhibitory in both the human prostate (PrCSC) and pancreatic (PCSC) cancer stem cells, with significant effects observed at early as 24 hr after infection, and progressing up to 72 hr (Figure 1A). The non-targeted lentiviral vector (sc-shRNA) generated modest but reproducible effects on cell growth over time, as we have observed in prior reports [45-47]. Cytotoxic effects of PKCδ depletion on the PCSC and PrCSC cultures were assessed by quantitating release of cellular LDH. Significant cytotoxicity was elicited by the PKCδ-specific shRNA as early as 24 hr after infection, with LDH release approaching the maximum possible levels by 72 hr. The effects of the scrambled shRNA on LDH release did not differ from those of the infection vehicle alone at any time point (Figure 1B). Efficient knockdown of the PKCδ isozyme was verified by immunoblotting (Figure 1C).

**Figure 2:**
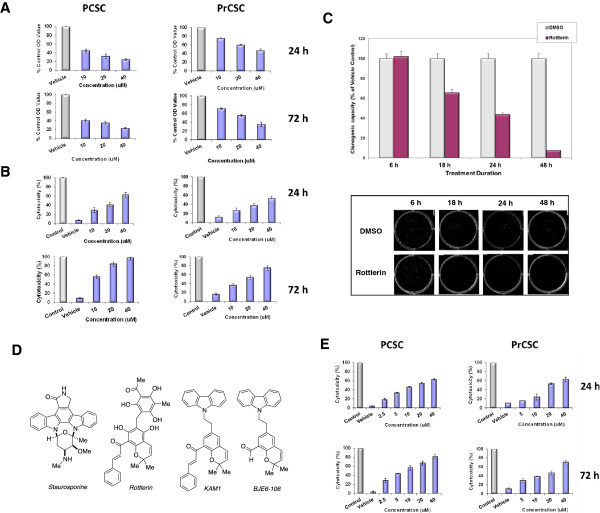
**Effects of PKCδ inhibitors on human cancer stem cells. (A)** PCSC and PrCSC cells at 80% confluence were exposed to rottlerin. DMSO served as vehicle control (Vehicle). After 24 and 72 hr of exposure, cell mass was evaluated by MTT assay. Control values were normalized to 100%. p values for comparison between treatments reached significance at 24 hr of exposure (p≤0.01) for both cell types, and remained significant at 72 hr. **(B)** PCSC and PrCSC cells at 50% confluence were exposed to rottlerin. Cytotoxicity was evaluated by LDH-release assay. Total maximal LDH release was assigned the arbitrary value of 100% (Control). p values for comparison between effects of treatments on LDH release reached significance at 24 hr of exposure (p<0.01) for both cell types, and remained significant at 72 hr. **(C)** Effects of PKCδ inhibitor on tumor cell clonogenic capacity. PCSC were exposed to vehicle or rottlerin (10 μM) for 6, 18, 24, or 48 hr. Viable cells were enumerated and re-plated in media without inhibitor, and colony numbers were quantitated 15 days later. p values for comparison of treatment effects on clonogenic capacity reached significance (p=0.005) at 18 hr of exposure and remained significant for all subsequent exposure times. The insert is a photograph of stained colonies on plates. **(D)** Structures of staurosporine, rottlerin, second-generation (KAM1) and third-generation (BJE6-106) derivatives. **(E)** PCSC and PrCSC cells at 50% confluence were exposed to KAM1 at the indicated concentrations. DMSO served as vehicle control (Vehicle). Cytotoxicity was evaluated by LDH-release assay, as in panel B. p values for comparison between treatment effects on LDH release reached significance at 24 hr of exposure to 2.5 μM KAM1 for PCSC cells and at 10 μM for PrCSC (p≤0.01), and remained significant at 72 hr for all concentrations of KAM1. Error bars represent SEM.

While the specificity of shRNA is essential for validation of a target, small-molecule enzyme inhibitors are more likely than shRNA to translate towards clinical application. We therefore next examined the effects of existing and novel small molecule inhibitors of PKCδ. Rottlerin, a natural product, has been identified as a PKCδ inhibitor for many years [47], with an *in vitro* IC_50_ of approximately 5 μM in our kinase assays (Table 1), in good agreement with the literature [57,58] (although it also exerts inhibitory effects on certain non-PKC kinases at concentrations comparable to the IC_50_ for PKCδ [59]). We and others have shown that rottlerin, at the concentrations employed herein, is not cytostatic or cytotoxic to normal primary cells or cell lines, and is well-tolerated when administered orally or intraperitoneally to mice (see also the studies on normal human breast epithelial cells and the *in vivo* studies later in this report) [45-47]. Exposure of PCSC and PrCSC cultures to rottlerin produced a significant dose-dependent inhibition of proliferation as early as 24 hr after exposure (Figure 2A). Similarly, rottlerin induced cytotoxicity in both CSC cultures in a dose-dependent fashion, as assessed by LDH release (Figure 2B). The duration of PKCδ inhibition required to irreversibly prevent CSC proliferation was next assessed. Exposure to rottlerin efficiently decreased the clonogenic capacity of PCSC. Eighteen hr of exposure to rottlerin, followed by washout, was sufficient to decrease the clonogenic capacity of PCSC by 40%, and increasing the duration of the exposure to 48 hr reduced the clonogenic potential by more than 90% (Figure 2C).

**Table 1:** Comparison of three generations of PKCδ inhibitors

**Generation**	**PKCδIC**_**50**_	**PKCαIC**_**50**_	**PKCδ/PKCα**
			**Selectivity ratio**
1	3 μM	75 μM	28-fold
2	2 μM	157 μM	56-fold
3	0.05 μM	50 μM	1000-fold

As previously reported, we have sought to develop novel PKCδ-inhibitory molecules with greater specificity for PKCδ compared to essential PKC isozymes, such as PKCα, using pharmacophore modeling and structure-activity relationships (SAR) [47]. We designed and synthesized a set of analogs based on this strategy. In this 2^nd^ generation of PKCδ inhibitors, the “head” group (carbazole portion) was made to resemble that of staurosporine, a potent general PKC inhibitor, and other bisindoyl maleimide kinase inhibitors, with two other domains (cinnamate side chain and benzopyran) conserved from the rottlerin scaffold to preserve isozyme specificity. The first such chimeric molecule reported, KAM1 (Figure 2D), was indeed active, like staurosporine, but was also more PKCδ-specific, and showed potent activity against Ras-mutant human cancer cells in culture and *in vivo* animal models, while not producing cytotoxicity in non-transformed cell lines [47]. KAM1 induced cytotoxicity as assessed by LDH release in a dose-dependent fashion in both PCSC and PrCSC cultures at concentrations as low as 2.5 μM (PCSC) and 5 μM (PrCSC) (Figure 2E).

On the basis of SAR analyses of KAM1, we then designed thirty-six new 3^rd^-generation analogs. The synthetic chemistry platform that was used to prepare KAM1 was modified to synthesize these additional analogs, which were then tested for biochemical and cellular activity. The PKCδ-inhibitory activity and isozyme-specificity of this 3^rd^ generation was quantitated *in vitro.* A number of these 3^rd^ generation analogs demonstrated significant increases in potency and isozyme specificity over rottlerin (1^st^ generation) and KAM1 (2^nd^ generation). The new compound selected for study in this report, BJE6-106, is much more potent than rottlerin. BJE6-106 has an (*in vitro*) PKCδ IC_50_ in the range of 0.05 μM, compared to 3 μM for rottlerin (Table 1), is approximately 1000-fold more inhibitory against PKCδ than PKCα *in vitro*, and produces cytotoxic activity against cells with aberrant Ras signaling at nM concentrations [55].

**Figure 3:**
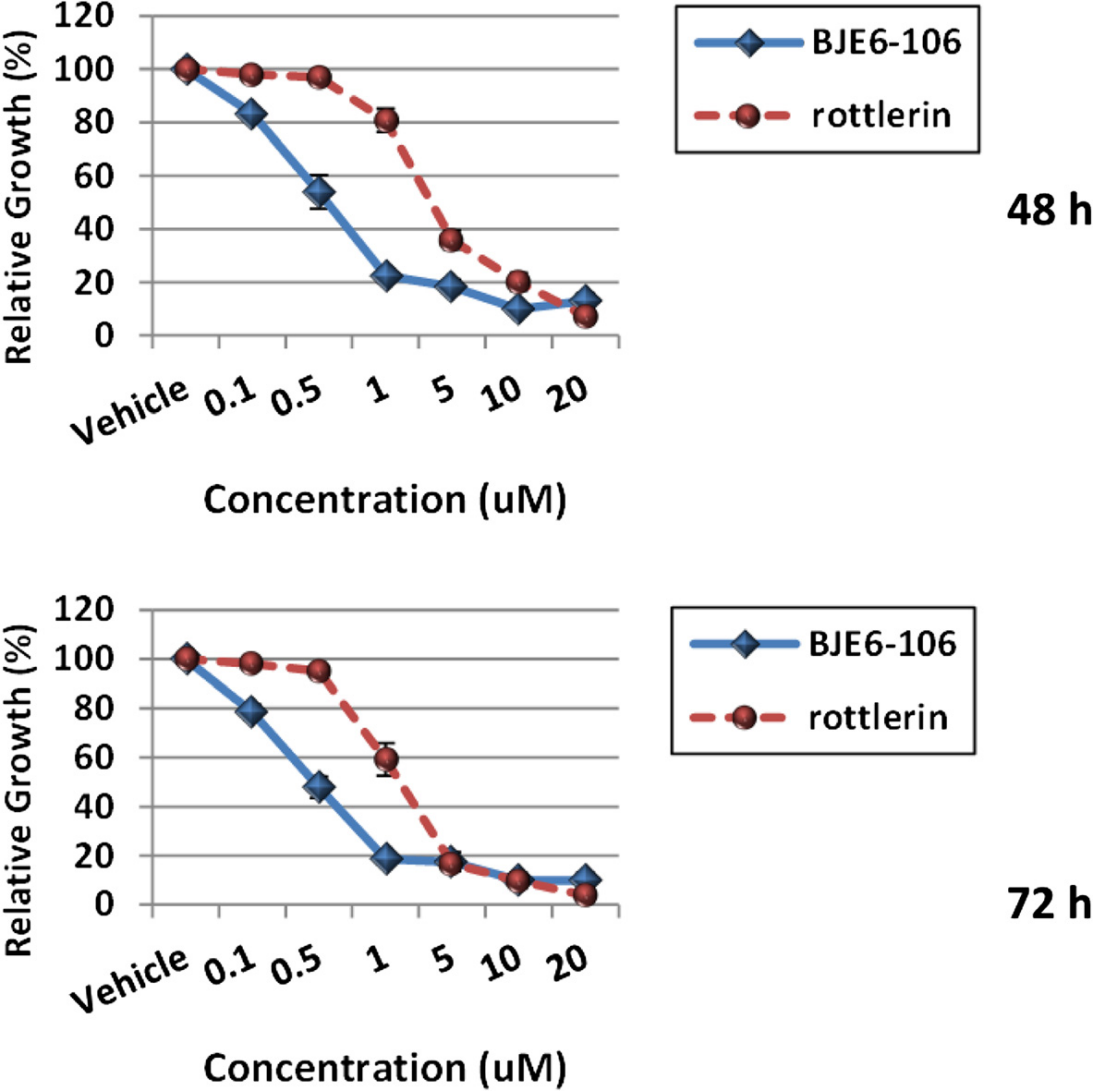
**Effects of a 3**^**rd **^**generation small molecule PKCδ inhibitor on human pancreatic cancer stem cell cultures.** PCSC cells were grown to 80% confluence in 96-well plates and then exposed to BJE6-106 at concentrations ranging from 0.1 to 20 μM, or to rottlerin at concentrations ranging from 1 to 20 μM. The corresponding equivalent volume of solvent (DMSO) was used as a vehicle control (Vehicle). After 48 and 72 hr of exposure, cell mass was evaluated by MTT assay. Control values were normalized to 100%. Error bars represent SEM. p values for comparison between vehicle and rottlerin effects on cell number at 48 hr reached significance at 1 μM, and for BJE6-106 at 0.1 μM (p ≤ 0.02), and remained significant at the 72 hr time point.

The activity of the 3^rd^ generation PKCδ inhibitor BJE6-106 on the growth of PCSC cells in culture was compared to rottlerin. BJE6-106 inhibited the growth of PCSC cultures at concentrations as low as 0.1 μM, and had an (in culture) IC_50_ of approximately 0.5 μM at 48 hr (Figure 3). In contrast, rottlerin produced no significant inhibitory activity at 0.5 μM, and displayed an IC_50_ at 48 hr of approximately 3 μM. LDH release assays also showed greater than 10-fold increases in potency for BJE6-106 compared to rottlerin (data not shown).

### Inhibition of PKCδ prevents tumor sphere formation

**Figure 4:**
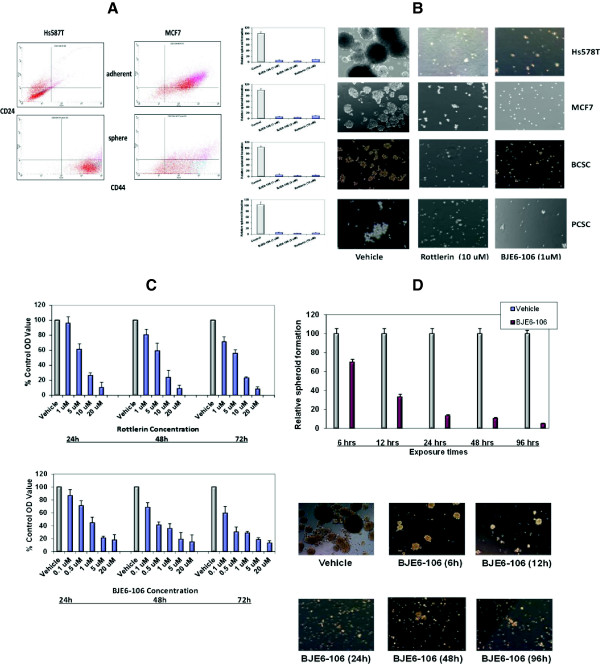
**Effects of PKCδ inhibitors on human tumor cell spheroid formation. (A)** Hs578T and MCF7 were plated under adherent or non-adherent conditions. Tumor spheroids and adherent cells were collected at 96 hr, stained for CD24 and CD44, and analyzed by flow cytometry. **(B)** Hs578T, MCF7, breast cancer stem cells (BCSC) and pancreatic cancer stem cells (PCSC) were plated in tumor spheroid media, in the presence of rottlerin, BJE6-106, or DMSO (Control). Tumor spheroids were enumerated at 96 hr, and normalized to the number of spheroids in the control cultures (assigned an arbitrary value of 100%). p values for comparison between vehicle and rottlerin or BJE6-106 effects were significant (p≤0.001). Photographs are of representative areas of the culture plates. **(C)** MCF7 cells were exposed BJE6-106 or to rottlerin at the indicated concentrations. The corresponding equivalent volume of solvent (DMSO) was used as a vehicle control (Vehicle). After 24, 48 and 72 hr of exposure, cell mass was evaluated by MTT assay. Control values were normalized to 100%. p values for comparison between vehicle and rottlerin effects on cell number at 24 hr reached significance at 5 μM, and for BJE6-106 at 0.5 μM (p ≤ 0.02), and were significant for all concentrations tested at 48 and 72 hr time points. **(D)** Hs578T cells were exposed to vehicle or BJE6-106 (1 μM) for 6, 12, 24, 48 or 96 hr. Viable cells were enumerated and re-plated in media without BJE6-206, and spheroid numbers were quantitated 96 hr later. p values for comparison between vehicle and BJE6-106 effects on spheroid number were significant after 6 hr of exposure (p≤0.02), and remained significant at all time points thereafter. Error bars represent SEM.

Sphere formation assays, which have been commonly used to identify and purify normal and malignant stem cells, were used to select a “CSC-like population” from established human breast cancer cell lines Hs578T, MDA231 and MCF7. A subpopulation of these cell lines could grow as non-adherent spheres and be continuously propagated in a defined serum-free medium *in vitro*. Flow cytometry and immunofluorescence analysis indicated that sphere-derived cells from cell lines contained a much larger proportion of cells expressing CD44, a candidate surface marker of breast cancer stem cells, and/or a smaller proportion of cells expressing the non-stem cell marker CD24, compared with adherent cells (Figure 4A). The frequency of spheroid formation relative to input cell number was low for the tumor cell lines (≤2-3%), as expected. In contrast, spheroid formation from the cultures of primary PCSC or primary breast cancer stem cells (BCSC) was much more efficient (45% and 53%, respectively). As expected, the CD24/CD44 profiles of cells in the spheres derived from the primary PCSC and BCSC did not differ from the adherent cells (not shown).

Addition of rottlerin or BJE6-106 to the culture medium very efficiently inhibited the formation of spheroids from all of these cell types (Figure 4B), demonstrating cytostatic or cytotoxic activity on tumor cells having a CSC-like phenotype. Interestingly, the actions of these compounds appeared to be even more potent on the CSC subpopulation in the MCF7 cell line than on the adherent “parental” cells (although different assays are being compared). When the MCF7 adherent population, containing predominantly non-CSC, was exposed to rottlerin or BJE6-106, concentrations in excess of 10 μM and 1 μM, respectively, were required to repress growth by more than 80% (Figure 4C). In contrast, growth of MCF7 spheroids was inhibited greater than 90% by rottlerin at 10 μM and BJE6-106 at 1 μM. Washout studies using spheroid formation demonstrated that as little as 6 hr of exposure to BJE6-106 at 1 μM significantly repressed spheroid formation of Hs578T cells, with near maximum inhibition achieved by 24 hr of exposure (Figure 4D).

In parallel studies, BJE6-106 at 0.5-1.0 μM and rottlerin at 10 μM also efficiently inhibited the growth of tumor spheroids generated from two human melanoma cell lines (SBcl2, >99.5% inhibition, p < 0.001; FN5, >99.5% inhibition, p < 0.001), two human pancreatic cancer cell lines (MiaPaCa2, >97% inhibition, p < 0.001; Panc1, >99% inhibition, p < 0.001); and two prostate cancer cell lines (DU145, >98% inhibition, p < 0.001; PC3, >96% inhibition, p < 0.001).

**Figure 5:**
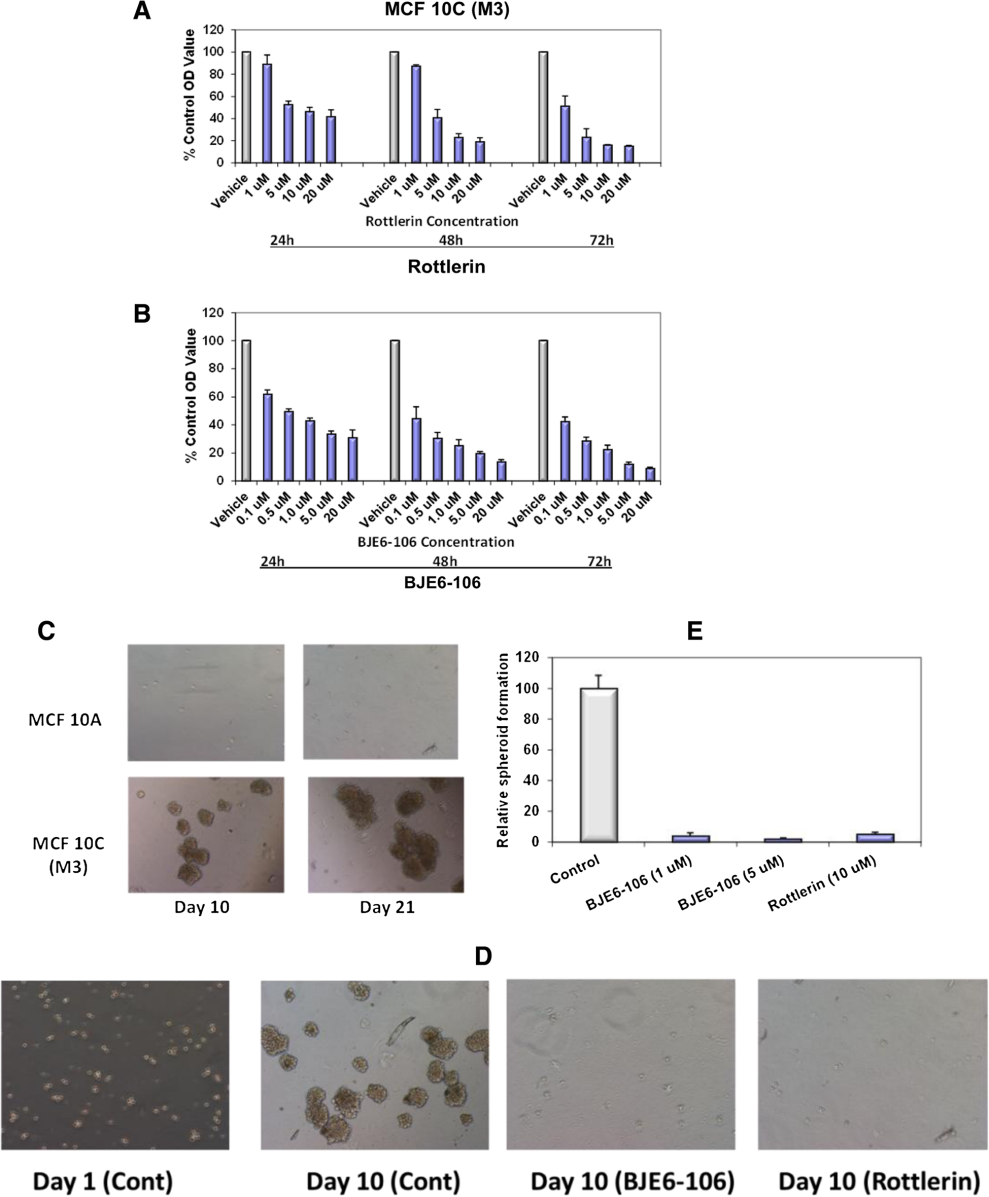
**Effects of PKCδ inhibitors on growth and spheroid formation in non-transformed and transformed human breast epithelial cells.** MCF 10A cells and cells from the derived tumorigenic line MCF 10C (also called M3), were grown to 80% confluence in 96-well plates and then exposed to rottlerin at concentrations ranging from 1 to 20 μM **(A)** or to BJE6-106 at concentrations ranging from 0.1 to 20 μM **(B)**. The corresponding equivalent volume of solvent (DMSO) was used as a vehicle control (Vehicle). After 24, 48 and 72 hr of exposure, cell mass was evaluated by MTT assay. Control (vehicle) values were normalized to 100%. Error bars represent SEM. p values for comparison between vehicle and PKCδ inhibitors on MCF 10A cell number only reached significance (p < 0.05) at 48 hr at 20 μM for rottlerin, and at 1 μM for BJE6-106. In contrast, significant effects of the inhibitors on the MCF 10C cells were observed as early as 24 hr for rottlerin (at 5 μM) and for BJE6-106 (at 0.1 μM). **(C)** MCF 10A and MCF 10C cells were plated at 10,000 cells per well in tumor spheroid media, and spheroid formation was assessed at days 10 and 21. Representative photographs are shown. **(D)** MCF 10C cells were plated at 10,000 cells per well in tumor spheroid media, in the presence of rottlerin (5 μM), or BJE6-106 (1 μM or 5 μM), or DMSO vehicle (Control). Tumor spheroids were enumerated at 10 days. Representative photographs are shown. **(E)** Spheroid numbers were normalized to the number of spheroids in the control cultures (assigned an arbitrary value of 100%) and plotted. Error bars represent SEM. p values for comparison between vehicle and rottlerin or BJE6-106 effects on spheroid number were significant (p < 0.001).

A CSC-like phenotype can be induced during epithelial-mesenchymal transition (EMT) in transformed cell lines. Transformation of the “normal” human mammary epithelial cell line MCF 10A and selection for a tumorigenic, metastatic phenotype *in vivo* produced the derivative line MCF 10C [53,54], which exhibits an EMT phenotype [60]. Cells of this malignant derivative also became ALDH + [61]. Transformation of these cells rendered them sensitive to rottlerin (Figure 5A) and to BJE6-106 (Figure 5B), compared to the parental MCF 10A line. The IC_50_ of rottlerin and BJE6-106 for the MCF 10C derivative was approximately 1 μM and 0.1 μM, respectively, at 72 hr, whereas the IC_50_ for the parental MCF 10A cells were >20 μM.

The MCF 10C derivative also acquired the ability to efficiently form non-adherent spheroids (Figure 5C), in contrast to the parental MCF 10A cells. Growth of these spheroids was efficiently inhibited by exposure to rottlerin at 10 μM or to BJE6-106 at 1 μM (Figure 5D and E).

The relative lack of toxicity of PKCδ inhibition on the non-transformed, “normal” breast epithelial MCF 10A cells is noteworthy, and further supports the established non-essential role of this isozyme in normal cells and tissues. In other work, we have demonstrated that normal mouse embryo fibroblasts and human primary fibroblasts and epithelial cells and microvascular endothelial cells and primary melanocytes survive and proliferate in the setting of PKCδ knockdown or in concentrations of PKCδ inhibitors which are lethal to tumor cell lines with aberrant Ras signaling ([45-47,55]; Trojanowska et al., in preparation).

### Inhibition of PKCδ inhibits CSC tumor xenograft growth

**Figure 6:**
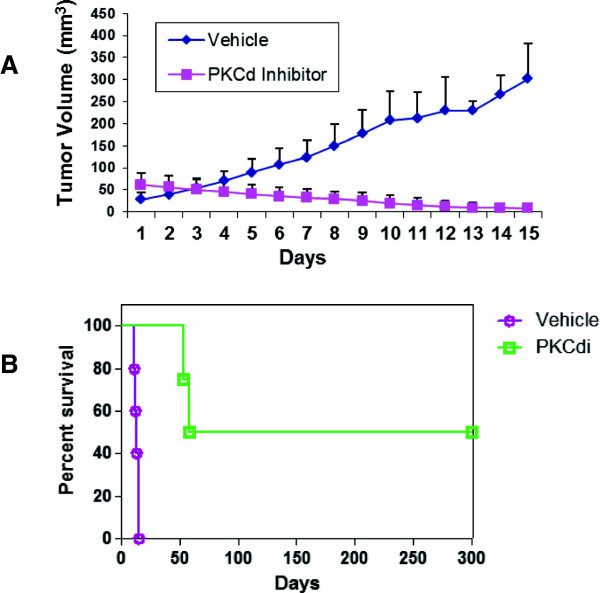
**Effects of PKCδ inhibitor on tumor growth and survival in a xenograft human breast cancer stem cell model.** Breast cancer stem cell xenografts were established and animals were treated with vehicle or rottlerin for 15 days, as described in Methods. **(A)** Tumor volumes plotted over time, until tumors in all the control animals reached the maximum volume allowed by the protocol (approximately 15 days). **(B)** Kaplan-Meier plot of survival of vehicle control or rottlerin (PKCδi)-treated animals, with monitoring continuing after cessation of treatment at day 15.

Another property of CSCs is their high tumorigenic potential. We therefore next sought to determine if PKCδ inhibition would inhibit the growth of CSCs *in vivo*. While the 3^rd^ generation PKCδ inhibitory compounds such as BJE6-106 are more potent and more cytotoxic to tumor cells and CSCs than previous generations, they have not been optimized for drug-like properties and are highly hydrophobic and poorly bioavailable, making efficient delivery of this generation of compounds *in vivo* unreliable. We therefore tested a prior-generation PKCδ inhibitor, rottlerin, which is readily bioavailable, in a tumor model. The human breast cancer stem cell (BCSC) cultures efficiently formed tumors as xenografts in nude mice. In comparison to vehicle control, rottlerin delivered intraperitoneally 5 days out of 7 effectively inhibited the growth of the xenografts, even producing tumor regression (Figure 6A). Survival was calculated on the day when tumor size reached the predetermined limit volume in the animals. The survival of the treated cohort extended long beyond the treatment interval, with some animals remaining tumor-free even at day 300 (Figure 6B).

**Figure 7:**
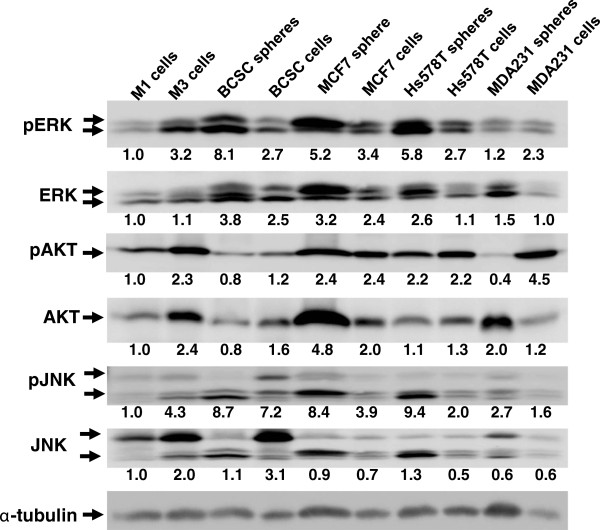
**Immunoblot analysis of Ras-signaling pathways in tumor cells, cell lines, or spheres.** Hs578T, MCF7, MDA231 and breast cancer stem cells (BCSC) were plated in tumor spheroid media under adherent or non-adherent conditions. Tumor spheroids and adherent cells were collected at 96 hr, and lysed. MCF 10A (M1) and MCF 10C (M3) lysates were also prepared. The lysates were separated by electrophoresis and immunoblotted with antibodies against ERK, pEKR, AKT, pAKT, JNK, pJNK. Immunoblotting of α-tubulin serves as a loading control. For quantitation, the digital intensity of the bands was first normalized to α-tubulin in each lane, and then expressed relative to the signal for the MCF 10A (M1) cell line. Values are shown under each band.

We have previously demonstrated that depletion of PKCδ is selectively toxic for cells with aberrant activation of Ras or Ras signaling pathways. Of the cell lines and CSC studied in this report, only a minority bore activating mutations of Ras itself (the pancreatic cancer cells are K-Ras mutant, and the melanoma cells are N-Ras mutant). MCF7 and the primary prostate and breast cancer stem cells, for example, had normal Ras alleles. Analysis of Ras signaling pathways of cells derived from the CSCs, however, showed relative increases of pERK or pAKT, compared to the respective parental (adherent, non-spheroid) cells (Figure 7). These findings indicate relative activation of the MEK/ERK pathway in BCSC, MCF7 and Hs578T CSCs, and relative activation of the PI_3_K-AKT pathway in MDA231 CSCs.

## Discussion

Small populations of cancer cells within multiple types of solid tumors have been identified based on cell surface marker expression and other phenotypic and functional characteristics. These subpopulations of tumor cells have often demonstrated a >100-fold increase in tumorigenic potential, compared to the remainder of the cells in the tumor. Furthermore, tumors that form from these cancer stem cells are indistinguishable from the human tumors in which they originate, indicating that the tumor-initiating cells are stem cell-like in their ability to self-renew and give rise to a heterogeneous cell population. Much recent data suggests that elimination of these cancer stem cells, which are typically resistant to conventional therapies, represents the most formidable barrier to curing solid tumors [1,4,5,32,33,35]. CSCs, or subclones thereof, are the most likely perpetrators of invasion and metastasis [6,62].

Recent findings have shown the existence of activated and quiescent repertoires of stem cells in established tumor cell lines as well as primary tumor cell isolates, and their ability to interchange between these conditions [37]. Sphere-forming assays (SFA) are believed to evaluate the differentiation and self-renewal capabilities of a tumor cell population by assessing the potential of a tumor cell to behave like a stem cell, and are widely used in stem cell studies [37]. Sphere-forming assays have been commonly used to retrospectively identify normal and cancer stem cells, and measure stem cell/early progenitor activity in multiple types of solid cancers [38,63,64]. Increased expression of “stemness-related genes” [65] was observed when comparing solid tumor cell lines grown as 3D spheroids to monolayers.

Our identification of PKCδ as a critical mediator of survival in multiple types of solid tumors, including prostate, breast, lung, pancreatic, neuroendocrine and melanomas [45-48] raised the possibility that CSC populations might be similarly dependent upon the activity of this enzyme. The effects of PKCδ inhibition on CSCs, however, had not been previously explored.

We first validated PKCδ as a target in diverse CSCs by demonstrating here that specific and selective down-regulation of PKCδ by shRNA was sufficient to prevent the growth of human breast, pancreatic and prostate cancer stem-like cell cultures, and to induce cytotoxicity.

Potential therapeutic translation of this synthetic lethal approach required the development of small molecule probes. As no PKCδ-selective inhibitors had been developed to date, we initially used pharmacophore modeling and docking of rottlerin, a well-established but not highly-specific inhibitor of PKCδ, into the crystal structure of PKCθ, to identify regions of the molecule important for PKCδ-selectivity. The initial new molecule showing activity against PKCδ (KAM1) was formed by combining structural elements of the broad spectrum protein kinase inhibitor staurosporine and rottlerin. The chromene portion of rottlerin was combined with the carbazole portion of staurosporine to produce KAM1 [47]. KAM1 was further modified to develop 36 new analogs, including BJE6-106, which inhibits PKCδ with an IC_50_ value of 50 nM and is approximately 1000-fold selective versus PKCα. Specificity for PKCδ over “classical” PKC isoforms, like PKCα, is important, as inhibition of PKCα is generally toxic to all cells, normal and malignant, and would render these inhibitors non-“tumor-targeted”. We have shown that B106 exerts potent cytotoxic activity against N-Ras-mutant human melanomas and B-Raf-mutant melanoma lines that have developed resistance to B-Raf inhibitors by aberrant activation of alternative Ras signaling pathways [48,55].

We demonstrate here that first, second and third generation PKCδ inhibitors (exemplified by rottlerin, KAM1 and BJE6-106, respectively), inhibit the growth of human cancer stem-like cell cultures isolated from tumors, as well as CSC-like cells derived from cell lines by spheroid formation on non-adherent surfaces. Our prior studies would have predicted that the CSC isolates or spheroids derived from cell lines that contained activating mutations of N-Ras or K-Ras would likely be susceptible to PKCδ suppression (*e.g.*, the K-Ras mutant pancreatic carcinomas and the N-Ras mutant melanomas). The reason for the susceptibility of the stem-like tumor cells containing wt-Ras alleles, however, was not immediately apparent. One reason for their susceptibility is likely to be upregulation of Ras effector pathways (MEK-ERK or PI_3_K/AKT signaling) in CSC spheres derived from cell lines, compared to the non-CSC parental cultures. We have reported previously that isolated activation of the MEK-ERK effector pathway or the PI_3_K/AKT effector pathway was sufficient to make cells dependent upon PKCδ for survival [45-47]. The finding of higher levels of Ras effector pathway activation in the CSC sphere subpopulation compared to the parental cells may also explain why in at least one instance (MCF7) the sphere-forming CSC cells were substantially more susceptible to PKCδ inhibition than non-CSC cells population. Interestingly, a recent report has identified a requirement for PKCδ in erbB2-driven proliferation of breast cancer cells [66], and erbB2 drives aberrant Ras pathway signaling. Furthermore, activation of MAPK pathways in basal-like breast cancers has been reported to promote a cancer stem cell-like phenotype [67], and activation of Ras/MAPK signaling was reported to protect breast cancer stem cells from certain stem-cell targeted drugs [68]. Collectively, these reports, together with our findings, suggest that a PKCδ-targeted approach to breast cancer stem cell populations, which exploits a synthetic lethal interaction with aberrant Ras signaling, may be particularly effective.

Inhibitory effects of PKCδ suppression on the IL6-Stat3 axis, which is critical for CSC genesis or maintenance in a number of tumor cells types [69-71], may also contribute to the actions of PKCδ inhibition on CSC growth and survival, and will be reported separately.

Epithelial-to-mesenchymal transition (EMT), induced either by paracrine signaling from cancer-associated fibroblasts (CAFs) or neighboring tumor cells, has been associated with the acquisition of a stem cell phenotype [72]. In culture, when immortalized normal or transformed human mammary epithelial cells (HMECs) are stimulated to undergo an epithelial-to-mesenchymal transition (EMT), the transition confers stem-like cell properties upon normal or transformed epithelial cells in culture, partly because the cells acquire a CD44+/CD24 (low) phenotype, similar to breast cancer stem cells.

The idea that cancer cells might reversibly transition between epigenetically-defined tumorigenic and non-tumorigenic states is of interest in part because mechanisms that generate reversible heterogeneity can confer resistance to therapies [73,74]. We took advantage of a previously-established cell line model system for breast cancer EMT, which consists of a parental spontaneously-immortalized mammary epithelial cell line, MCF 10A (M1), and one of its derivatives, MCF 10C (M3), derived from a xenograft in nude mice that progressed to carcinoma [53,54]. These cell lines were previously reported to exhibit distinct tumorigenic properties when re-implanted in nude mice; MCF 10A is non-tumorigenic, while MCF 10C forms low-grade, well-differentiated carcinomas [53,54,60]. Furthermore, MCF 10C has acquired phenotypic changes consistent with mesenchymal morphology and gene and protein expression patterns characteristic of EMT, including expression of mesenchymal markers (fibronectin, vimentin, and N-cadherin) with concomitant downregulation of E-cadherin, β-catenin, and γ-catenin. MCF 10C also expresses high levels of Nanog, and Sox4, which are markers of cancer stem cells [61]. We found that the mesenchymal, CSC-like MCF 10C subline was much more sensitive to PKCδ inhibitors than the epithelial-like “normal” MCF 10A cells from which they were derived. Furthermore, the MCF 10C line acquired the capacity to efficiently form spheroids when grown in non-adherent conditions, and this tumor spheroid formation was inhibited by inhibition of PKCδ activity.

## Conclusions

Collectively, these findings suggest that human cancer stem-like cells isolated from diverse sources and tumor types require PKCδ activity for their growth or maintenance *in vitro* and *in vivo*, making this isozyme a novel tumor-specific target. Taken together with the previous demonstration by our group and others of the cytotoxic effects of PKCδ inhibition on the non-CSC population of many tumor cell types, PKCδ inhibitors hold the promise of eliminating both the majority non-CSC population and the latent and resistant CSC population comprising human tumors.

## Abbreviations

BCSC: Primary human breast adenocarcinoma stem cells; CSC: Cancer stem-like cell; MAPK: MAP kinase; PCSC: Primary human pancreatic adenocarcinoma stem cells; PKCδ: Protein kinase C delta; PKCα: Protein kinase C alpha; PrCSC: Primary human prostate adenocarcinoma stem cells; shRNA: Short hairpin RNA

## Competing interests

DVF and RMW have applied for a patent on certain of the PKC-delta inhibitory compounds described in this report. The other authors have no competing interests to disclose.

## Authors’ contributions

ZC and LWF carried out the molecular and biochemical studies, and participated in the preparation of the manuscript. RMW and DVF designed the novel inhibitory compounds. RMW synthesized the compounds and participated in the preparation of the manuscript. DVF conceived the study, and participated in its design and coordination and drafted the manuscript. All authors read and approved the final manuscript.

## Pre-publication history

The pre-publication history for this paper can be accessed here:

http://www.biomedcentral.com/1471-2407/14/90/prepub
